# Whole-Genome Sequences of Three Edwardsiella piscicida Isolates from Diseased Fish in South Korea

**DOI:** 10.1128/mra.00097-22

**Published:** 2022-04-25

**Authors:** Nameun Kim, Yoonhang Lee, Ho Diem Tho, Ji Yeon Park, Ju Yeop Lee, Yu-Ra Kang, Do-Hyung Kim

**Affiliations:** a Department of Aquatic Life Medicine, Pukyong National University, Busan, Republic of Korea; University of Arizona

## Abstract

Edwardsiella piscicida is a Gram-negative pathogen that is associated with edwardsiellosis in aquaculture systems worldwide. Here, we report the whole-genome sequences of three E. piscicida isolates derived from cultured fish in South Korea.

## ANNOUNCEMENT

Previous studies (1, 2) revealed that Edwardsiella tarda encompassed three genetically distinct taxa, namely, E. tarda, Edwardsiella piscicida, and Edwardsiella anguillarum. Recent investigations showed that E. piscicida was one of the most important bacterial pathogens in farmed fish (3, 4). Although Edwardsiella ictaluri is the most common bacterial pathogen in catfish (5, 6), a previous study (7) showed that E. piscicida was more virulent than E. tarda or E. anguillarum in channel catfish. In this study, we report three E. piscicida strains, which were isolated from diseased olive flounder (Paralichthys olivaceus) (two strains) and Korean catfish (Silurus asotus), using hybrid assemblies of Nanopore and Illumina reads.

Strains KE5 and 18EpOKYJ, which were isolated from diseased olive flounder in 1999 and 2018, respectively, and strain CZ12001, which was isolated from diseased catfish in 2012, were collected from fish farms in South Korea. All strains were identified as E. piscicida using the species-specific primers developed in a previous study (6). A single colony of each E. piscicida strain grown on tryptic soy agar (Oxoid, Thermo Fisher Scientific, USA) was inoculated into Luria-Bertani broth (Oxoid) and incubated at 28°C for 18 h. Genomic DNA was extracted using the Wizard DNA purification kit (Promega, USA) and quantified using a Qubit v3.0 fluorometer (Thermo Fisher Scientific). The Illumina sequencing library was constructed using the Nextera DNA Flex library preparation kit (Illumina, USA) and Nextera DNA CD indexes (Illumina). Sequencing was performed on an iSeq 100 system (Illumina) using 150-bp paired-end reads. Long reads were generated on a MinION sequencer (Oxford Nanopore Technologies [ONT], UK) using a FLO-MIN106 flow cell (ONT), and a library was constructed using the one-dimensional ligation sequencing kit (SQK-LSK109; ONT) and barcoding kit (EXP-NBD104; ONT), following the manufacturer’s protocol. Fast5 reads were generated, and base calling and demultiplexing were conducted using MinKNOW v19.10.1 software (ONT) with a high-accuracy base-calling mode.

FastQC v0.11.9 (8) and MinIONQC v1.4.2 (9) were used to evaluate sequence quality. A hybrid Nanopore-Illumina *de novo* assembly was performed using Unicycler v0.4.9 (10) in the normal assembly mode, including a polishing step with Pilon. The circularity of all contigs was confirmed by Unicycler. The assembly quality was assessed using Benchmarking Universal Single-Copy Orthologs (BUSCO) v5.2.2 (11) (data set: lineage enterobacterales_odb10) and QUAST v5.0.2 (12). Genome annotations were carried out with Prokka v1.14.5 (13). The average nucleotide identity (ANI) values were determined using fastANI v1.33 (14), in comparison with E. piscicida ET883^T^ (GenBank accession number JRGQ00000000), E. tarda ATCC 15947^T^ (GenBank accession numbers CP084506 to CP084509), E. anguillarum ET080813^T^ (GenBank accession numbers CP006664 to CP006666), and E. ictaluri ATCC 33202^T^ (GenBank accession number AFJI00000000). Default parameters were used for all software unless otherwise specified.

The sequencing and annotation results are summarized in Table 1. A single circular chromosome was confirmed by Unicycler for all isolates. A unique single circular plasmid, with a GC content of 51%, was found in strain 18EpOKYJ. The strain CZ12001 genome was composed of five contigs, including contig 1 as a circular chromosome (3,832,345 bp) and four contigs, with circular forms varying in size from 90,212 bp to 10,711 bp, as plasmids with GC contents of 53% to 48%. The ANI values for KE5, 18EpOKYJ, and CZ12001 with respect to the E. piscicida type strain (ET883^T^) were all >99%, but those for our strains with respect to E. tarda ATCC 15947^T^, E. ictaluri ATCC 33202^T^, and E. anguillarum ET080813^T^ were approximately 84%, 92%, and 95%, respectively. *In silico* analysis revealed that CZ12001 harbored type IV secretion system gene clusters in the plasmid.

**TABLE 1:** Summary of genome data for the isolates

Strain	Illumina sequencing		ONT sequencing			No. of contigs	Total length (bp)	GC content (%)	Complete BUSCOs (%)	No. of protein-coding genes
	No. of reads	Coverage (×)^*a*^	No. of reads	*N*_50_ (bp)	Coverage (×)^*a*^					
KE5	1,596,130	64	148,841	15,650	257	1	3,763,397	59.73	97.7	3,273
18EpOKYJ	1,928,134	76	148,228	19,336	433	2	3,819,771	59.61	97.7	3,338
CZ12001	1,483,432	55	238,076	20,433	623	5	4,058,165	59.29	97.9	3,552

a Rounded to the nearest whole number.

### Data availability.

Raw sequences of both Illumina and Nanopore reads were submitted to NCBI SRA with accession numbers SRR14845009 to SRR14845014 under BioProject number PRJNA738669 and BioSample numbers SAMN19736599, SAMN19736600, and SAMN19736601. The assembled genome sequences of the three Edwardsiella piscicida isolates were deposited in GenBank with accession numbers CP090962 to CP090969.
