# Correction for Kim et al., “Whole-Genome Sequences of Three *Edwardsiella piscicida* Isolates from Diseased Fish in South Korea”

**DOI:** 10.1128/mra.00892-22

**Published:** 2022-10-17

**Authors:** Nameun Kim, Yoonhang Lee, Diem Tho Ho, Ji Yeon Park, Ju Yeop Lee, Yu-Ra Kang, Do-Hyung Kim

## ERRATUM

Volume 11, no. 5, e00097-22, 2022, https://journals.asm.org/doi/10.1128/mra.00097-22. Page 1: This article was published on 25 April 2022 with Diem Tho Ho’s name rendered as Ho Diem Tho. The byline should appear as shown in this Erratum.

